# Supplementary material for: Diabetes Is an Independent Risk Factor for Cancer after Heart and/or Lung Transplantation
Source: J Clin Med. 2022 Jul 15;11(14):4127. doi: 10.3390/jcm11144127 (PMC9323113; doi:10.3390/jcm11144127)

Supplementary Material

Supplementary Table S1: Detailed pre-transplantation screening protocol.

Supplementary Table S2: Transplanted patients follow-up protocol.

Supplementary Table S3: Immunosuppression regimen and the standard plasma levels.

Supplementary Figure S1A and S1B: Graphical representation of covariate balance for the variables age and mycophenolate therapy.

Table S1. Detailed pre-transplantation screening protocol.

Requested Exams and Interdisciplinary Consultations
**Requested Exams**
Chest x-ray
Mammography (if older than 40 years)
Coloscopy (if older than 40 years)
Gastroscopy
Doppler (legs / pelvis)
Doppler of the carotids
Chest - computer tomography
Abdomen- computer tomography
Head and sinuses -computer tomography
Lung function (if necessary- 6 minute walk test)
Arterial blood gas analysis with indoor air breathing
Central venous blood gas analysis
ECG, possibly long-term ECG
Echocardiography
Spiroergometry
Left heart catheter
Right heart catheter with determination of pulmonary vascular resistance and cardiac output
Heart muscle biopsy (for cardiomyopathy)
Bronchoscopy
**Interdisciplinary Consultations**
Otorhinolaryngology
Ophthalmology
Dentistry
Gynecology / Urology
Dermatology
Nephrology
Psychological evaluation

Table S2. Transplanted patients: follow-up protocol.

Months after surgery	1	2-3	4-6	7-9	10-12	13-18	>18
Clinical appointment	weekly	every 2 weeks	every 3 weeks	every 4 weeks	every 6 weeks	every 10 weeks	every 12 weeks
Blood sample collection	weekly	every 2 weeks	every 3 weeks	every 4 weeks	every 6 weeks	every 6 weeks	every 6 weeks

Table S3A. Immunosuppression regimen.

**Timepoint**	Intraoperative	6h after surgery	Day 1 after surgery	Day 2 after surgery	Up to day 3 after surgery
**Medicament**					
Methylprednisolone (intravenous)	1000 mg	250 mg every 12 h (3 times)			
Prednisolone (intravenous)			0,5 mg/kgKG (3/5-0-2/5)	0,5 mg/kgKG (3/5-0-2/5)	2,5 mg reduction every 3 days
Tacrolimus (oral)			2 x 2 mg	2 x 2 mg	
Mycophenolate (oral)			2 x 1000 mg	2 x 1000 mg	2 x 1000 mg

Table S3B. Immunosuppressive standard plasma levels.

**Timepoint**	0-6 Months	6-12 Months	Up to 1st year	Up to 2nd year
**Plasma level (µg/l)**				
Mycophenolate	1,5 - 3	1,5 - 3	1,5 - 3	1,5 - 3
Tacrolimus	10 - 12	8 - 10	7 - 10	7 - 10
Ciclosporin	200 - 220	150 - 200	100 - 150	Under 100
Sirolimus	-	-	8 - 10	8 - 10
Everolimus	5 - 8	5 - 8	5 - 8	5 - 8

Figure S1A. Balance plot for the variable "age" before and after matching.

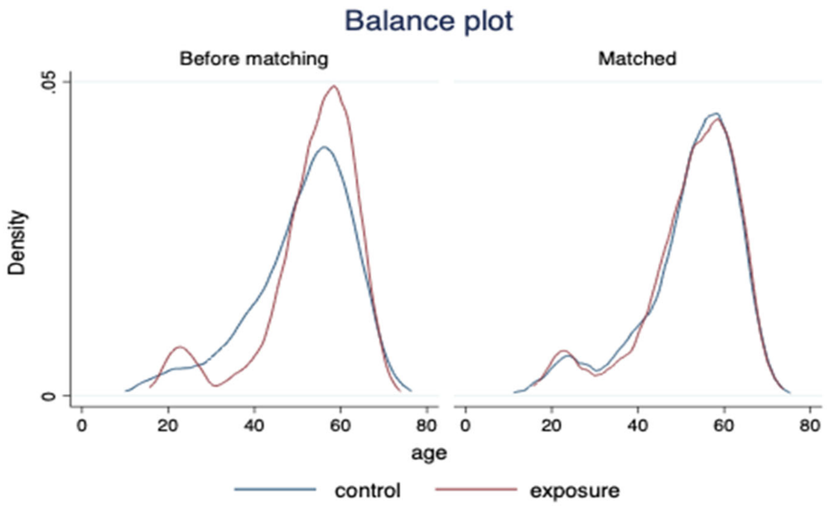

Figure S1B. Balance plot for the variable “mycophenolate therapy” before and after matching.

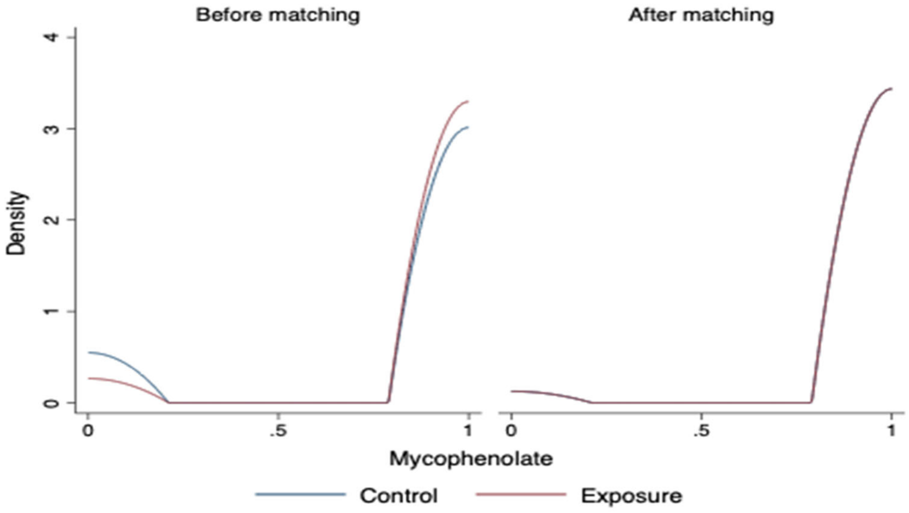

Supplement: Supplementary file 1 [file jcm-11-04127-s001.zip › jcm-1815506-supplementary.pdf]
